# Carbonic Anhydrases in Photosynthesizing Cells of C3 Higher Plants

**DOI:** 10.3390/metabo9040073

**Published:** 2019-04-16

**Authors:** Lyudmila Ignatova, Natalia Rudenko, Elena Zhurikova, Maria Borisova-Mubarakshina, Boris Ivanov

**Affiliations:** Institute of Basic Biological Problems, Federal Research Center – Pushchino Scientific Center for Biological Research of the Russian Academy of Sciences, Pushchino 142290, Russia; lkign@rambler.ru (L.I.); rudenko_n@rambler.ru (N.R.); zhurikova-alena@yandex.ru (E.Z.); mubarakshinamm@gmail.com (M.B.-M.)

**Keywords:** carbonic anhydrase, C3 plants, arabidopsis, gene expression, photosynthesis, chloroplasts, thylakoids

## Abstract

The review presents data on the location, nature, properties, number, and expression of carbonic anhydrase genes in the photosynthesizing cells of C3 plants. The available data about the presence of carbonic anhydrases in plasma membrane, cytoplasm, mitochondria, chloroplast stroma and thylakoids are scrutinized. Special attention was paid to the presence of carbonic anhydrase activities in the different parts of thylakoids, and on collation of sources of these activities with enzymes encoded by the established genes of carbonic anhydrases. The data are presented to show that the consistent incorporation of carbonic anhydrases belonging to different families of these enzymes forms a coherent system of CO_2_ molecules transport from air to chloroplasts in photosynthesizing cells, where they are included in organic molecules in the carboxylation reaction. It is discussed that the manifestation of the activity of a certain carbonic anhydrase depends on environmental conditions and the stage of ontogenesis.

## 1. Introduction

The process of CO_2_ incorporation into organic matter in higher plants takes places in chloroplasts, the specialized organelles of photosynthesizing cells containing thylakoids, flattened bubbles bound by a membrane where the ATP and NADPH-producing (during the light-dependent reaction stage of photosynthesis) photosynthetic electron transport chain (PETC) is located. The stroma, the space between the chloroplast envelope and thylakoids, contains water-soluble Calvin cycle enzymes responsible for light-independent reactions resulting in organic substance synthesis. Ribulose-1, 5-bisphosphate carboxylase/oxygenase (Rubisco), which makes up to 30% of cellular soluble protein, is the enzyme catalyzing CO_2_ incorporation into the organic molecule ribulose bisphosphate (RuBP). The second most abundant cellular protein (1–2%) is the water-soluble carbonic anhydrase, which is also located in the chloroplast stroma.

Carbonic anhydrase (CA) is the enzyme that catalyzes the reaction of reversible hydration of CO_2_ to HCO_3_^−^, increasing greatly the rates in both directions; the *k*_cat_ can be up to 10^6^ times higher as compared with spontaneous reaction constant, with larger acceleration of the hydration reaction. These enzymes are widely distributed in living systems and present in all cells of organs and tissues of animals, including humans, as well as in ascomycetes, bacteria, algae and higher plants. The first reference to the existence of the CO_2_-dependent enzyme was made in the work of Henriques in 1928, however the enzyme was called ‘carbonic anhydrase’ only later in 1933 [1]. After purification of the enzymes, it was demonstrated that CAs are zinc-containing enzymes [1,2]. Significant progress has been achieved in understanding the structure and function of CAs in animal cells, where they play a significant role in the kidney, pancreas, red blood cells and platelets, lungs, eyes, etc. [3,4,5]. The basic explanation of the importance of functioning of CAs is the integration of the enzymes into various processes involved in regulation of pH, CO_2_ and HCO_3_^−^ concentrations and their transport, as well as water and electrolyte balance [6]. All CAs are divided into several families based on conserved nucleotide sequences [7]. It is noteworthy that all known CAs in the vertebrate animals belong to the α-family.

Neish [8] was the first to report CA activity in whole leaves as well as in chloroplasts of several species of higher plants. Thus far, it has been determined that higher plants contain CAs belonging to α, β, and γ families [9]. Arabidopsis genome analysis identified 19 genes encoding CAs. The names for CAs of *Arabidopsis thaliana* used later are according to [10].

CAs of photosynthesizing cells excited interest primarily because the chloroplast stroma containing Rubisco has a slightly alkaline pH, which further increases to pH 7.7–7.8 when exposed to light. At these pH values, more than 95% of inorganic carbon is in HCO_3_^−^ form, and to provide for a high rate of CO_2_ fixation, a high rate of conversion of HCO_3_^−^ into CO_2_ is required, which is not possible in the case of a spontaneous reaction. Jumping ahead, we can point out that the role of the most abundant stromal CA still remains open.

CAs were attributed not only the role of ensuring the required conversion rate of HCO_3_^−^ molecules into CO_2_, but that of transporting inorganic carbon from air to the chloroplast stroma where the Rubisco is located. CAs were thought to be involved in facilitating the CO_2_ penetration through both the plasma membrane and chloroplast envelope membrane at the expense of fast interconversion of CO_2_ and HCO_3_^−^ on both sides of the membranes. It has been demonstrated that CA presence on both sides of the artificial membrane ensures quick CO_2_ diffusion through it [11]. Based on existing evidence, one can infer that the aggregate of higher plant photosynthesizing cell CAs should make up a coordinated functional system that ensures the delivery of inorganic carbon from the air to the carboxylation centres. Moreover, recent data suggest that CAs also play an important role in the reactions taking place in PETC. In this review, we will consider data on CA presence in C3 plant photosynthesizing cell structures, as well as the proven and proposed functions of these CAs.

## 2. CA Gene Expression in Arabidopsis Leaves

Gene expression of all β family CAs has been demonstrated in *Arabidopsis thaliana* leaves [10,12], as well as the expression of three α family genes encoding α-CA1, α-CA2 and α-CA3 [10]. Rudenko et al. [13] did not detect expression of the latter, but demonstrated that arabidopsis leaves contain *At4g20990* gene transcripts encoding α-CA4 found among thylakoid membrane proteins [14]. In arabidopsis, there are 6 active *β-ca* genes, and excepting *β-ca3* the others have from 2 to 5 forms, resulting from alternative splicing predominantly in untranslated regions of one or two exons by the 3’ ends, and more often, by the 5’ ends of the corresponding RNAs. Wang et al. [12] have shown that arabidopsis leaves contain virtually all of these RNA forms, except for *AtβCA2.3*. A study by Rudenko et al. [13] has determined that the expression intensity of two *β-ca1* forms, which differ from each other in exon splicing on the 3’ side, changed differently as plants adapted to increased illumination.

Expression intensity of most β-family CAs is several times higher than gene expression levels of α-CAs both in various organs of *Sorghum bicolour*, a C4 plant, [15], and in leaves of *A. thaliana,* a C3 plant [13]. These CAs have been studied more extensively than α-CAs due to considerably higher content of *β-ca* gene transcripts, as well as higher content of the corresponding proteins. Expression levels of CA genes change depending on the age of the plant and the growth conditions. These changes are highly dependent on the length of the photoperiod [13]: long-day (16 h day / 8 h night) and short-day (8 h day/16 h night) plants of the same age are in different ontogenetic stages as day length exceeding 12 h is the main factor contributing to the transition of arabidopsis into the generative phase. This transition is accompanied not only by a stronger expression in groups of genes responsible for inducing flowering, but by changes in the intensity of expression of many genes that are not directly related to flower organ development, likely including CA genes. Active transcription of all genes of β-family CAs, as well as *α-ca1*, *α-ca2* and *α-ca4* genes is present in leaves of adult 40–60 day-old arabidopsis plants grown in short-day conditions. By day 55, genes *β-ca2, β-ca4, β-ca5* tended only to increase in expression intensity, whereas the content of *β-ca3*, *β-ca6*, *α-ca2* gene transcripts increased considerably. Under long-day conditions, which are more similar to those under which the plants grow in nature, a decline in expression of most CA genes occurred directly before the plants transitioned to reproductive organ development [13].

## 3. Carbonic Anhydrase in Plasma Membrane

It is evident that the flow of CO_2_ molecules from air into the photosynthesizing cells is driven by the ‘disappearance’ of these molecules as they are fixed in chloroplasts in the light, generating a concentration gradient. CO_2_ molecules enter the leaf through the stomata and dissolve in the apoplast, an aqueous space surrounding photosynthesizing leaf cells. The slightly acidic conditions in the apoplast (pH 5.2–6.4 [16]) ensure that a goodly proportion of inorganic carbon within it is in CO_2_ form. The proportion of bicarbonate is lower at more low pH since the effective pK_1_ of carbonic acid, which decomposes into CO_2_ and water directly after forming is equal to 6.35: H^+^ + HCO_3_^−^ ↔ H_2_CO_3_ ↔ CO_2_ + H_2_O [17]. CO_2_ concentration in pure water at 25 °C is approximately equal to 10.2 µM. Due to a higher solubility in the membrane lipid layer compared to water, CO_2_ should freely diffuse through this lipid layer [18] into the cytoplasm with pH 7.6, where it can quickly convert into bicarbonate, aided by cytoplasmic CAs (see below). The membrane, however, is not just a layer of lipids; a considerable amount of protein at its boundaries may hinder the flow of CO_2_ through the membrane.

The presence of CA in the plasma membrane was first discovered during a study of the influence of CO_2_ concentration on CO_2_-dependent O_2_ evolution by photosynthesizing cell protoplasts in pea leaves [19]. This evolution, which correlates with photosynthesis rate, decreased when CO_2_ at the high concentration of 0.5 mM was introduced into a medium with protoplasts. This outcome resulted from an indirect transfer of protons, which appear during the hydration of CO_2_ molecules entering the protoplast [20], and from a shift in pH of chloroplast stroma to values below those optimal for the Calvin cycle. It was discovered that acetazolamide, a specific CA inhibitor with poor penetration into membrane, lowered the above mentioned O_2_ evolution suppression. This indirect evidence of CA presence in plasma membrane has been proven by detecting CA activity in these membranes of other C3 plants, both in dicotyledons and monocotyledons [21].

CA activity of whole intact protoplasts, which may be considered as plasma membrane CA activity, amounted to 3–11% of lysed protoplast CA activity. Calculations where plasma membrane volume was assumed to represent 0.3% of total protoplast volume have shown that the density of CA molecules on the plasma membrane is quite high [19,22]. It has been found that the affinity of plasma membrane CA to CO_2_ (K_0.5_ (CO_2_) = 104 mM) is much lower than that of stromal CA (K_m_(CO_2_) = 20 mM) [23]. Such low affinity of the plasma membrane CA to CO_2_ may be a remembrance of times when CO_2_ concentration in the Earth’s atmosphere was high.

**Figure 1:**
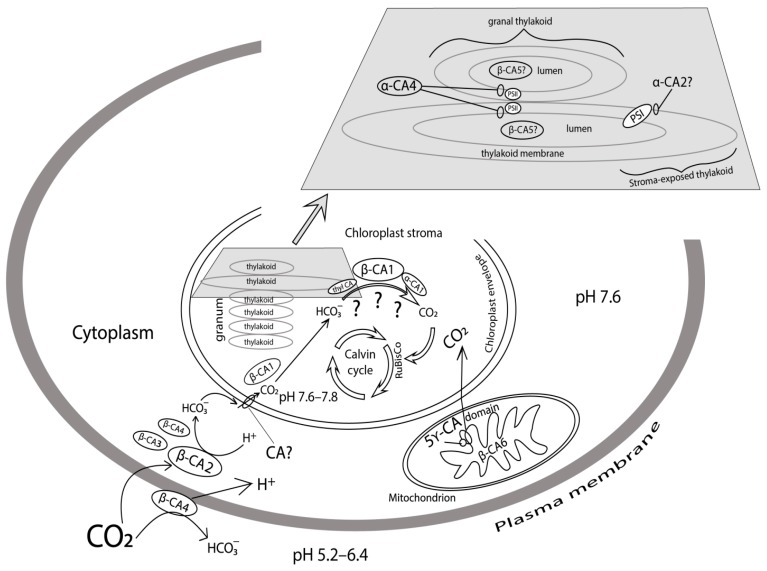
Schematic presentation of CA locations in the photosynthesizing cell of higher C3 plants.

The functioning of the plasma membrane CA in pea protoplasts was manifested not only at those high CO_2_ concentrations but also at concentrations near 100 μM. At this concentration saturating photosynthesis, the light-dependent proton uptake, the kinetics of which coincided with that of CO_2_-dependent O_2_ evolution at pH 7.2 was inhibited two-fold by 1 mM acetazolamide [24]. In that study, based on the dependencies of the effect on pH of medium, the scheme of CA operation in plasma membrane was proposed. It was assumed that this CA executed hydration of CO_2_ in the membrane, directing additional protons into cytoplasm where their appearance was not accompanied by the simultaneous formation of bicarbonate, as in the case of CO_2_ entry (Figure 1). This mechanism also explained the above described effect of acetazolamide at the high CO_2_ concentrations.

Fabre et al. [10] have demonstrated that *A. thaliana* plasma membrane contains products of the gene encoding β-CA4 by inserting a green fluorescent protein (GFP) gene. Later, DiMario et al. [25] discovered that one β-CA4 form is present in arabidopsis plasma membrane, while another form of this CA is present in the cytoplasm. Content of *β-ca4* gene transcript in short-day conditions did not depend on light intensity. In long-day plants, when plants receive more light, the content was approximately two times lower at high light intensity than at low intensity. It has not yet been possible to determine the specific function of this CA, and as in the case of other CAs, its function may manifest itself only under certain conditions; for example, when CO_2_ concentration in air increases to a considerable degree.

## 4. Carbonic Anhydrases in Cytoplasm

CAs have been detected in leaf cell cytoplasm in both C3 and C4 plants [26,27]. These water-soluble enzymes belong to the β-family, as it was determined later [28]. In C4 plant leaves, the highest CA activity was found in cytosol of mesophyll cells where the occurrence of a specific reaction of C4 photosynthesis, namely, the carboxylation of phosphoenolpyruvate using bicarbonate by phosphoenolpyruvate carboxylase, the primary carboxylase of C4 plants, requires the prompt conversion of atmospheric CO_2_ to bicarbonate, and cytosolic CA executes this conversion. This CA plays an important role because mutants with less than 10% of wild-type CA activity demonstrated very low CO_2_ assimilation rates and a depressed size at ambient CO_2_ [29]. In bundle sheath cells of C4 plants of three types, however, very low CA activities or their absence were reported, and it was noted that the presence of CA might reduce the efficiency of CO_2_ fixation by Rubisco in chloroplasts of these cells [30].

CA presence in the slightly alkaline cytoplasm of photosynthesizing cells of C3 plants also contributes to a faster conversion of CO_2_ molecules, which have diffused through the plasma membrane into bicarbonate ions, and helps new CO_2_ molecules to enter the cytoplasm (Figure 1). However, this formation of HCO_3_^−^ is only one out of several stages in the process of inorganic carbon supply for photosynthesis in chloroplasts. In C3 plants, for example in potato leaves, cytoplasmic CA activity accounts for 13% of the total CA activity in the leaf [31]. Two isoforms of β-family CAs were found in *A. thaliana* leaf cell cytoplasm using the GFP fusion method: β-CA2 and β-CA3, with β-CA3 appearing to be the less abundant cytoplasm protein [10]. Fett and Coleman [32] have previously described the extrachloroplastic CA in pea. This CA was called CA2, and it was presumed to be situated in the cytoplasm. Fabre et al. [10] have determined that the gene product in arabidopsis leaves they denoted as β-CA2 corresponded to that abundant CA2. This study has shown that the content of *β-ca3* transcripts is much lower than that of *β-ca2* using semi-quantitative PCR [10]. In another study [13], a quantitative PCR was used and it was determined that *β-ca3* gene expression was, depending on the growing conditions, 3–5 orders of magnitude lower than that of *β-ca2,* which qualitatively complied with data obtained by Fabre et al. [10].

The impact of light intensity on cytoplasmic CA gene expression was strongly dependent on ontogenetic stage. During a long-day (16 h) photoperiod *β-ca2* expression intensity did not depend on light intensity, while during a short day (8 h) 14 days after plants were transferred to high illumination conditions *β-ca2* expression intensity increased several times, with *β-ca3* expression intensity declining after illumination increased both under short-day and long-day conditions [13]. The authors of the study [13] have proposed that both cytoplasmic CAs take part in inorganic carbon supply to the cell, but their contributions to the process vary, depending on the growing and development conditions of the plants. This cooperation of two CAs can be illustrated by the high-expression of *cah1* gene, induced by low ambient CO_2_ concentrations, and the extremely low-expression of *cah2*, induced by high ambient CO_2_ concentrations, functioning within the *Chlamydomonas reinhardtii* periplasmic space [33], both of which participate in delivering inorganic carbon to the cell.

DiMario et al. [25] have recently shown that a third CA, one of the β-CA4 forms, is present in *A. thaliana* cytoplasm. The presence of products of three genes of CAs in the cytoplasm suggests that there is likely a need to regulate not only inorganic carbon supply into the cell, but the conversion of inorganic carbon forms during multiple processes taking place in this compartment, such as, for example, amino acid biosynthesis.

## 5. Carbonic Anhydrases in Mitochondria

Five structurally linked subunits representing CAs of γ-family have been detected in higher plant mitochondria, where they form a spherical domain that is a part of complex I of the respiratory chain [34]. Moreover, a matrix localized CA of β family, β-CA6 has been found in *A. thaliana* mitochondria [10]. Figure 1 shows the positions of these CAs.

Utilization of CO_2_ evolved in the course of respiration in mitochondria for fixation in chloroplasts is believed to take place in higher plants. Mitochondrial CAs are clearly significant for preserving CO_2_ generated in mitochondria during photorespiration, as it has been found that both photorespiration inhibitors and ethoxyzolamide, a sulphonamide CA inhibitor, suppressed photosynthesis of protoplasts of mesophyll cells at limiting CO_2_ concentrations [35]. Zabaleta et al. [36] supposed that the domain of five CAs promoted bicarbonate formation followed by its transfer from mitochondria to chloroplasts. This export system may be important in conditions promoting intensification of the photorespiration, such as high light or high temperature. In order to propose a targeted mechanism for transporting inorganic carbon from mitochondria to chloroplasts, it would be necessary to assume that these organelles come into specific contact. Chloroplasts and mitochondria are actually often found in contact with each other in the cell, but more likely, the inorganic carbon, which is released in the mitochondria during respiration, enters the chloroplasts through the concentration gradient created by CO_2_ consumption in the course of its fixation. 

Soto et al. [37] proposed that β-CA6 together with the γ-CA domain participate in the system of transporting inorganic carbon from the mitochondria to the chloroplasts when the stomata are closed and its uptake from the environment is limited. In this case β-CA6 activity should be induced by low carbon dioxide concentrations, yet Wang et al. [12] did not demonstrate any changes in *β-ca6* transcription intensity when carbon dioxide concentrations in air increased or decreased, with transcription intensity increasing significantly 48 hrs after plants had been in the dark [12]. It can be assumed that there is no immediate relationship between the functioning of the β-CA6 and photosynthesis since although its overexpression in *A. thaliana* resulted in an increase in plant biomass there was no significant change in photosynthetic rates compared to wild type (WT) plants [38]. The fact that the content of gene transcripts encoding β-CA6 did not depend on light intensity is also likely to be evidence of the lack of this relationship [13]. Mitochondrial β-CA6 in higher plants may perform the same role as CAs CAH4 and CAH5 in the *Chlamydomonas reinhardtii* mitochondrial matrix, which, as shown in the study by Giordano et al. [39], catalyze the conversion of carbon dioxide formed in the tricarboxylic acid cycle into bicarbonate, which is later used in the NH_4_^+^ assimilation reaction, the products of which are used in protein synthesis.

## 6. Carbonic Anhydrases in Chloroplasts

### 6.1. Carbonic Anhydrases in Chloroplasts Stroma

The carbonic anhydrase localized in the chloroplast stroma is denoted in *A. thaliana* as β-CA1 and, as already noted, is the most abundant leaf CA. Stromal CA has been known for several decades and is the most studied plant CA, yet, strange as it may seem, there is no consensus on its main function in chloroplasts of C3 plants. It has been proposed a long time ago that this CA assists in CO_2_ diffusion across the chloroplast envelope to provide the necessary rate of CO_2_ supply for carboxylation of RuBP in Calvin cycle [40]. The function of the CA in this case is acceleration of conversion of CO_2_ entering the stroma into bicarbonate ions in alkaline medium, which becomes more alkaline in the light (Figure 1). More frequently, the main function was believed to be to enable the conversion of bicarbonate into CO_2_, a substrate for Rubisco incoming directly to this enzyme. However, it was discovered that mature tobacco [41] and *A. thaliana* [42] plants with highly reduced levels of this CA, produced by means of antisense or knockout showed no changes in photosynthetic activity.

These results also appear to dismiss a proposed mechanism in which this CA takes part in increasing the local availability of CO_2_ for Rubisco [43]: it had been suggested that since carboxylation of RuBP leads to two protons appearing in the medium, the equilibrium between CO_2_ and bicarbonate at the site where these protons are formed should shift towards CO_2_, and the CA that is in direct contact with Rubisco accelerates this shift. However, CA and Rubisco complexes, as well as the inclusion of CA in a supramolecular protein complex consisting of Rubisco and other enzymes of the Calvin cycle have been discovered in pea and tobacco [44,45,46], serving as evidence of the above mechanism.

One more soluble CA, α-CA1, has been found relatively recently in *A. thaliana* chloroplast stroma [47]. There is one study that demonstrates a decrease in photosynthetic activity and starch accumulation capacity in mutant plants defective in the gene encoding that CA [48]. The expression level of the *α-ca1* gene was significantly lower than that of *β-ca1* under all growing conditions [13]. The same study considered the influence of increased illumination of plants on the expression of two forms of the gene *β-ca1* and the gene *α-ca1*. After an initial drop, on the third day of acclimation to high light intensity the content of transcripts of both CA genes increased, and after 14 days of high light it was several times higher than in plants that continued to grow under low light intensity. The data from these studies [13,48] suggest that α-CA1 does play a role in photosynthesis, but as *α-ca1* gene expression did not depend on carbon dioxide concentration during plant growth [10], it is likely required, just like β-CA1, for processes that take place in the chloroplast stroma and intensify with an increase in photosynthesis rate under increasing plant illumination.

In addition to the above-mentioned functions that the stromal CA may perform in photosynthesis, the roles in other processes were also attributed to it. It undoubtedly participates in stabilizing pH in the stroma through a rapid scattering of excess protons or hydroxyl, which may be generated by biochemical processes in the stroma [40]. Moreover, stromal β-CAs of C3 plants have been shown to take part in pathogen resistance [49], seedling survival [42], and lipid biosynthesis [50]. Slaymaker et al. [51] have established that stromal CA demonstrates not only CA activity, but also a capability to bind salicylic acid, which plays an important role in signalling, setting off a cascade of reactions designed to protect plants from stress. In *A. thaliana*, stromal β-CA1 performs an important established function together with plasma membrane β-CA4 in stomata movements through a CO_2_-controlled signalling pathway [52].

As it has not been possible to unequivocally establish the involvement of soluble stromal CAs in the immediate CO_2_ supply to Rubisco, it can be suggested that, although they may participate in this process, stroma-oriented CAs of thylakoid membranes contribute to it the most. Figure 1 shows the possible ways of converting HCO_3_^−^ into CO_2_ with the participation of different CAs, and additional studies are required to prove each of them.

### 6.2. Carbonic Anhydrases in Thylakoids

#### 6.2.1. Carbonic Anhydrases in Thylakoid Membranes

For a long time, the consideration of the role of CAs in processes taking place in chloroplasts has been confined to studying their participation in soluble carbonic anhydrases located in the stroma of these organelles. Meanwhile, as early as the beginning of the 1980s, membrane-bound CA activity has been found in the chloroplast thylakoid membranes of bean [53] and pea [54]. However, for many years, the activity of thylakoid CA was not recognized, and it was considered to be a contamination of thylakoid membranes with highly active and abundant stromal CA. A. Stemler [55] has presented comprehensive analysis of facts evidencing the presence of the specific membrane-bound thylakoid CA. Multiple results have shown that the properties of membrane-bound thylakoid CA differ from those of soluble stromal CA: the redox state of the medium affected the CA activity of thylakoid membranes [56], the specific inhibitors of CAs, acetazolamide and azide, in sub-micro molar concentration had unusual stimulating effects on this activity [57], the pea thylakoid CA had higher affinity to CO_2_ than stromal CA, namely K_0.5_(CO_2_) of thylakoid CA was 9 mM whereas K_m_ of soluble stromal CA was 20 mM [23]. Moreover, dehydration activity of thylakoid CA depended on pH with maximum activity at 6.8–7.0, but the activity of soluble CA did not demonstrate pH dependence [23]. Finally, the antibodies against soluble CA from spinach demonstrated strong cross-reaction with soluble CA of pea chloroplasts, but not with thylakoids possessing similar CA activity [58].

At the beginning of the 2000s, the first evidence of the presence of more than one membrane-bound CA in thylakoids started to appear. The fragments of thylakoid membranes highly enriched with Photosystem II or Photosystem I (PSII- and PSI-membranes) from pea possessed CA activity, but only PSII-membranes demonstrated [59] cross-reaction with antibodies against CAH3, α-CA from *Ch. reinhardtii* that is attached to the thylakoid membrane from the lumenal side [60]. Incubation of thylakoids with progressively increasing amounts of Triton X-100 showed two distinct maxima of their CA activity at 0.3 and 1.0 Triton/chlorophyll ratios [61], evidencing the existence of at least two membrane-bound CAs within the thylakoids membranes. It is worthy of note that the maximum CA activity in PSI-membranes was registered at Triton/ chlorophyll ratio of 0.3, and in PSII-membranes at Triton/ chlorophyll ratio of 1.0, both in pea and arabidopsis [61,62].

PSI-membrane carbonic anhydrase from pea was equally sensitive to both specific inhibitors (acetazolamide and ethoxyzolamide) with I_50_ at 10^−6^ M [63], indicating that the enzyme active centre is on the surface of the thylakoid membrane. That said, CA activity of PSII-membranes was highly sensitive to ethoxyzolamide (I_50_ = 10^−9^ M), with acetazolamide at a low concentration stimulating that activity, and even a high content of this inhibitor did not fully inhibit CA activity of PSII-membranes from pea and arabidopsis [62,63,64].

It has recently been demonstrated that tri-fluoromethanesulfonamide (TFMSA), a specific CA inhibitor, decreased both the photo-induced changes of chlorophyll fluorescence yield and the photosynthetic oxygen evolution by specifically inhibiting CA activity of PSII-membranes [65]. This evidenced that CA activity was needed for maximum PSII activity. Such effects of known inhibitors, acetazolamide and ethoxyzolamide, were discovered long ago [66,67], but as it was later determined, the effects resulted, at least in part, from their ability to non-specifically suppress the electron transport rate in PSII [68,69]. By contrast with acetazolamide, TFMSA influenced the electron transfer rate in PSII only in HCO_3_^−^/CO_2_-free medium; adding exogenous bicarbonate, as well as electron donors for PSII, made the inhibiting effect of TFMSA disappear [65].

It was also shown that isolated pigment-protein complexes of PSII from maize mesophyll possessed two sources of CA activity with different properties [70]. The evidence in favour of the presence of two sources of CA activity in PSII-membranes was also obtained for pea [58,64] and for arabidopsis [62]. The CA activity appeared in solution after treating PSII-membranes either with salts in high concentration [58,70] or with detergents [64], but a significant part of the CA activity remained in PSII membranes [58,64,70,71,72]. The presence of two sources of CA activity in PSII was also revealed after native electrophoresis of detergent-treated PSII membranes from pea and arabidopsis: CA activity was detected in the gel band containing the low molecular mass proteins as well as in the upper gel area containing proteins associated with the PSII core complex [62,64,73]. The measurements of the activity of the eluates from these bands also supported the presence of CA activities in these bands [61,62].

Therefore, experimental results suggested that at least three CAs are present in thylakoid membranes. Various suggestions have been made regarding the nature of the sources of CA activity of thylakoids and their fragments. CA activity of PSII-membranes called ‘intrinsic’ in [70], or ‘high molecular mass’ in [62,64] related to components of the PSII core complex, is most likely not genuine CA activity, but rather a CA-like activity enabling bicarbonate supply to non-heme iron near the primary quinone acceptors of PSII. It is known that eliminating bound bicarbonate from thylakoid membranes leads to the termination of electron transfer at that site of the electron transport chain [74]. Mass spectrometry analysis has shown that proteins CP43 and CP47 of the PSII complex are present in a high molecular mass protein band with CA activity (Ignatova, L.K., unpublished).

As for another CA in PSII-membranes, which was called ‘extrinsic’ [70], or ‘low molecular mass’ [62,64], it has been suggested that PsbO, a manganese-stabilising protein of the oxygen-evolving complex, has CA activity [70,75]. This suggestion was oppugned [76], and it was later proven that the isolated protein PsbO did not demonstrate CA activity, and that the elimination of PsbO in PSII-membranes by treatment with salts did not lead to a decrease of their CA activity [77].

How is CA activity of thylakoid membranes and their fragments related to CAs encoded by specific genes found in the arabidopsis genome? α-CA4 was detected among arabidopsis thylakoid membrane proteins [14]. Its role in the processes that take place in these membranes was studied using a knockout mutant lacking gene *At4g20990* encoding this CA [78,79]. It was noted that higher magnesium content was required to isolate PSII-membranes from knockout plants than from wild type plants (WT), which is indicative of abnormal PSII complex structure. These mutants lost the above-mentioned (in this section) property related to stimulating CA activity by acetazolamide in sub-micro molar concentration characteristic of low molecular mass PSII CA [77]. Such stimulation of activity by azole inhibitors of CAs is specifically characteristic of α-family CAs [80]. Based on the above data, the location of α-CA4 in close vicinity to PSII complex is shown in Figure 1. 

Knockout of the gene encoding α-CA4 influenced arabidopsis growth and photosynthesis: the weight of above-ground parts of mutant plants and starch content were higher, and a higher amount of hydrogen peroxide was accumulated in leaves in light compared with WT [78,79]. In addition, CO_2_ assimilation rate and the extent of non-photochemical quenching (NPQ) of chlorophyll *a* fluorescence at high light intensity were lower than in WT [78,79,81]. The authors proposed that α-CA4 participates in protonation of PsbS protein located near PSII, which contributes to the progress of the energy-dependent part of NPQ, qE, from the first minutes of illumination [79,81]. This suggestion corresponds with a marked elevation in *At4g20990* gene expression in WT plants in high light, especially under long-day conditions [13] when the demand for NPQ, which protects PSII from photoinhibition is increased. It was established that the knockout of the gene encoding α-CA4 had an influence on the size of the light-harvesting antenna of PSII, with the content of the major antenna proteins, Lhcb1 and Lhcb2, lower when α-CA4 was absent than in WT plants under any growing conditions [81].

Photosynthesis characteristics in mutants with knocked out gene *At2g28210* encoding α-CA2 were studied in parallel to those in knockout mutants lacking the gene encoding α-CA4. The light-induced accumulation of hydrogen peroxide, the starch content in leaves of mutants deficient in α-CA2, and the effective quantum yield of PSII was lower, and NPQ and the CO_2_ assimilation rate were higher than in WT plants under the same growth and experimental conditions [79]. The contrasting effect on photosynthesis characteristics of the knockout of genes encoding α-CA4 and α-CA2 and a whole range of other characteristics of thylakoids from α-CA2 mutant made it possible to conclude that α-CA2 is localized in the thylakoid membrane, namely on its stromal side [82] (Figure 1). As for its function, it has been suggested that it adjusts the proton concentration in the thylakoid lumen, controlling proton outflow under stress conditions [82]. This is also evidenced by an increase in *α-ca2* gene expression under such stress conditions as increased light intensity and decreased CO_2_ concentration in the air [10,13].

#### 6.2.2. The Role of Carbonic Anhydrase in the Stimulation of Photophosphorylation by Bicarbonate

Recent evidence indicates that CA may participate in the stimulation of photophosphorylation (PP) by bicarbonate in thylakoid membranes of chloroplasts. This effect was discovered as early as 1964 [83], with later findings showing that HCO_3_^−^ also stimulates other types of energy-dependent ATP synthesis, namely, ADP phosphorylation after turning off light and phosphorylation initiated by an acid-base transition in the absence of light. The stimulation of PP by bicarbonate was attributed to its effect on the interaction between the coupling factor and the thylakoid membrane in an energized state [84], and the subsequent study revealed that the effect could be due to the change of the coupling factor conformation under change of membrane energization [85].

Studies [86,87] suggest that since adding HCO_3_^−^ to thylakoids with acetazolamide and ethoxyzolamide did not stimulate PP, CA should in some way participate in the above process. However, as mentioned earlier, acetazolamide and ethoxyzolamide may suppress electron transport, and therefore PP. In our research [69], the effect of bicarbonate was studied in isolated pea thylakoids, using mafenide, a hydrophilic CA inhibitor, which at concentrations that did not influence electron transfer and PP under control conditions, decreased the stimulation of PP by bicarbonate significantly. A number of facts both in previous works [83,84,85,87] and in [69] implied that the effect of HCO_3_^−^ is confined to the surface of thylakoid membrane. We proposed [69] a hypothetical mechanism of effect of bicarbonate addition to thylakoids that explained not only PP stimulation, but also the concurrent electron transport inhibition (even though under normal conditions, electron transport increases when the PP rate increases), and a higher effect in ammonium ion presence. The hypothesis was that the bicarbonate dehydration reaction is catalysed by a CA located on the surface of the thylakoid membrane, allowing a part of the CO_2_ molecules to inflow through this membrane into thylakoid lumen, where the hydration of CO_2_ molecules aided by luminal CA (see below) leads to an increase in proton concentration. Only these protons are involved in both the stimulation of PP and the inhibition of the electron flow along PETC.

#### 6.2.3. Carbonic Anhydrase in Thylakoid Lumen

A water-soluble CA was unexpectedly detected in a supernatant obtained after high-speed centrifugation of destroyed pea thylakoids, which had been thoroughly washed from stromal CAs [61]. Then, the same CA was detected in WT arabidopsis thylakoids, as well as in thylakoids of arabidopsis plants with knocked out gene encoding stromal β-CA1 [88]. The latter confirmed that the detected CA was not contamination with abundant stromal CA, and it was concluded that this CA is situated in the thylakoid lumen. The apparent molecular mass of the water-soluble CA of thylakoids was determined to be 262 kDa using native electrophoresis. The high molecular mass, the pattern of sensitivity to sulphonamide inhibitors, an increase in activity in the presence of dithiothreitol allowed attribution of this CA to the β-family [88].

Presumably, CA in the lumen is β-CA5 (Figure 1) detected in *A. thaliana* chloroplasts [10]. The transcription intensity only of gene *At4g33580* encoding β-CA5 increased when illumination decreased both in short-day and long-day conditions [13]. This dependence of *β-ca5* gene expression on plant illumination corroborates the suggestion that this gene encodes CA in lumen. CA in thylakoid lumen may enable a more free diffusion of protons to the ATP-synthase channel together with a CO_2_/HCO_3_^−^ buffer [87], and the value of such a diffusion should decrease at low light intensity when proton inflow into the lumen is low and they may be ‘lost’ on the way to the ATP-synthase.

Seedlings of mutant arabidopsis plants with knocked out gene *β-ca5* were well behind wild type plants in size [89]. This is the only case when knocking out just one CA has such a negative impact on the phenotype, and indicates that β-CA5 is the enzyme that is needed for normal physiological development of plants. 

## 7. Conclusions

While considerable progress has been made in understanding CA functions in such highly specialised animal tissues as blood and kidneys, and much has been achieved in identifying the functions of these enzymes in microalgae, progress in understanding the functions of CAs in photosynthesizing cells of higher plants where these functions appear obvious is rather slow. This may be caused by several concurrent circumstances that impede the understanding of functions of individual CAs. Unlike in animal cells, carbon dioxide is an indispensable substrate, and its consumption and use in photosynthesizing cells are vital to the plant. CAs are a part of the mechanism behind these complex processes, and their functional activity may remain undetected under conditions favourable for plant life when CO_2_ supply and fixation are not limited by the plant’s general metabolism at optimal light intensity, temperature, mineral nutrition, etc. Perhaps this is why we were able to detect the absence of one or another CA in mutants only after placing plants under stress conditions [78,79,81]. According to published data, metabolism impairment may occur under favourable conditions only in double CA mutants [52].

The complexity of studying CA functions in photosynthesizing cells of higher plants is compounded by the fact that multiple enzymes may be present in just one compartment, and that CAs, as we believe, operate as a system, which easily adapts to the absence of some elements. Sustainable functioning of these systems may be facilitated by the fact that unlike in the vertebrate animals where only CAs of α-family are present, CAs in higher plants belong to three enzyme families and have various molecular mass, pH optimum of operation, and binding with membrane. In some cases, as described in this review, the main function of CAs in photosynthesizing cells of plants is possibly not the conversion of CO_2_ into HCO_3_^−^ and vice versa, but rather using this conversion to maintain the required pH levels in various cell compartments. This CA function is not easily detected, and especially not under stress conditions because photosynthesizing cells contain many other buffer systems.

Using inhibitors, especially when identifying the functions of thylakoid CAs, is also complex as they may have a non-specific action on electron carriers of PETC localized in the thylakoid membrane. Therefore, it is likely impossible to explain the suppression of electron transport by CA inhibitors through them specifically inhibiting the activity of some thylakoid CAs.

It appears that further progress in understanding the functions of photosynthesizing cell CAs may be made by using both individual CA mutants and double mutants, as well as by applying new methods for tracking inorganic carbon transport inside the cell. It is also necessary to vary plant living conditions, as the significance of a CA for cell metabolism may manifest itself during a specific ontogenetic stage and under stress conditions.
